# Veterinary Legislation in New York

**Published:** 1895-02

**Authors:** 


### EDITORIAL.

As we go to press information reaches us that those opposed to the appointment of a veterinarian in executive capacity as a member of the Live Stock Sanitary Board in Pennsylvania have relinquished their opposition, and that a State veterinarian at a salary of $2500 per year will have a place in the new department of agriculture to be established as a State department in this State. This will be grateful news to every progressive veterinarian, and especially to the profession in Pennsylvania, for no concession was ever won under a more stubborn and unrelenting opposition.

